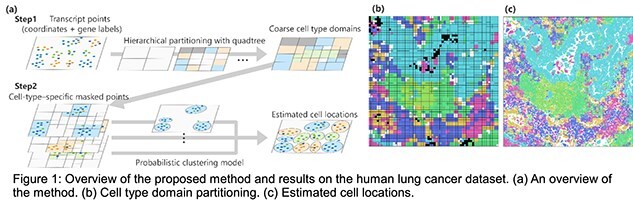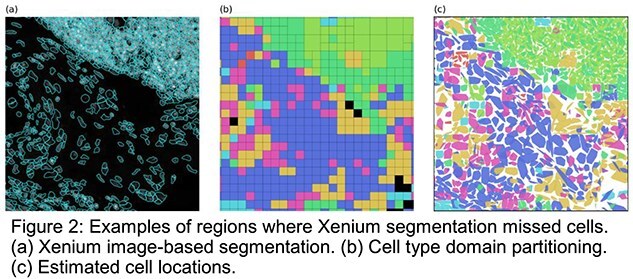# Image-free estimation of cell locations and types in spatial transcriptomics via quadtree partitioning and probabilistic clustering

**DOI:** 10.1093/bib/bbaf631.005

**Published:** 2025-12-12

**Authors:** Hibiki Sugiyama, Hironori Shigeta, Shunji Umetani, Shigeto Seno

**Affiliations:** The University of Osaka; The University of Osaka; The University of Osaka; The University of Osaka

## Abstract

**Introduction:**

Gene expression analysis is crucial for understanding cell identity and function. While single-cell RNA sequencing (scRNA-seq) successfully unveiled cellular heterogeneity, it critically loses the spatial context where cells reside and interact. To address this, spatial transcriptomics (ST) techniques have emerged. Among these, fluorescence *in situ* hybridization (FISH) based methods have garnered significant attention for their ability to quantify hundreds of genes with subcellular resolution. These methods generate rich transcript point cloud data that incorporates both spatial coordinates and gene identity.

A fundamental challenge in ST analysis is to assign each transcript to its corresponding cell to construct accurate cell-level gene expression profiles. Conventional methods often rely on the segmentation of stained images of the nuclei and membrane. However, these image-based approaches suffer from major limitations: low image quality, the need for labor intensive staining, and poor performance in densely packed or noisy tissue regions. Therefore, there is a need for a robust method that can estimate cell properties directly from transcript data without depending on image morphology.

In this study, we propose a method to estimate the positions and types of individual cells directly from transcript point clouds. Unlike conventional approaches, our method requires no auxiliary imaging and aims to provide reliable cell-level assignments in noisy or densely packed tissues.

**Proposed method:**

Our method estimates the locations and types of individual cells directly from the input transcript point cloud data, where each point is defined by its two-dimensional spatial coordinates and gene identity. Our approach relies on two important properties of tissue organization: tissues are hierarchically structured from cell types down to individual cells, and each cell type is characterized by a distinctive gene expression signature. Cells of the same type therefore form spatially coherent domains that can be distinguished by their expression profiles.

The approach consists of two steps. First, we partition the tissue space into hierarchical regions using a quadtree [1] to approximate coarse cell-type-specific domains. This enables the identification of transcript subsets likely belonging to a given type. Second, within each subset, we apply a probabilistic mixture model to identify individual cells. Each component is modeled by a two-dimensional Gaussian distribution for spatial positions and a categorical distribution for gene identities. The parameters and transcript assignments are estimated using an Expectation–Maximization (EM) algorithm. An overview of the proposed method is shown in Fig. 1 (a).

To distinguish cell types, our method requires reference data showing the gene expression pattern of each cell type, for example, annotated scRNA-seq data. The output is an assignment of transcripts to unique cell IDs and cell type labels. By directly leveraging spatial and gene expression signatures, our method eliminates the need for auxiliary images, ensuring effectiveness in noisy or densely packed tissues.

**Experiment:**

We evaluated our method on a human lung cancer dataset acquired with the Xenium platform [2]. Annotated scRNA-seq data [3] covering multiple cell types was used as reference data. As a baseline, we compared against the image-based segmentation provided by the Xenium Onboard Analysis pipeline [4].

Our results are shown in Fig. 1 (b) and (c), where hierarchical partitioning and clustering were used to define transcript assignments and cell regions. Furthermore, as illustrated in Fig. 2 (a), image-based segmentation failed to recognize cells in certain regions because of weak or ambiguous staining. In contrast, our method was able to reliably detect the cells in these regions (Fig. 2 (b), (c)). This confirms that our approach can robustly identify cells by leveraging transcript distributions alone.

Quantitatively, our method achieved a significantly higher transcript-to-cell assignment rate of 0.9996, compared to 0.7891 achieved by the Xenium segmentation. This substantial difference indicates that our method drastically reduces the number of unassigned transcripts and missed cells, providing a more complete profile of tissue heterogeneity. To assess the consistency of the identified cell boundaries, we treated both our unique cell assignments and the image-based segments as cell-level clustering. Their agreement yielded an Adjusted Rand Index (ARI) of 0.4653 and a Normalized Mutual Information (NMI) of 0.8939. These metrics show that our image-free method achieves a comparable level of agreement with the conventional image-based segmentation, while operating solely on transcript data.

Overall, the results demonstrate that our method achieves a substantially higher transcript assignment rate and comparable segmentation quality to image-based methods, confirming its robustness and potential as an effective, image-free alternative for cell identification in spatial transcriptomics.

**Discussion:**

Our study demonstrates the feasibility of estimating cell positions and types solely from transcript point clouds. By combining spatial organization with characteristic expression profiles, the method enables reliable transcript-to-cell assignment without auxiliary imaging. Applied to lung cancer data, it achieved a higher transcript assignment rate and strong agreement with image-based segmentation, while also detecting cells and transcripts missed by conventional methods. Future directions include scaling the model to larger datasets, validating it across diverse tissue types, and automating processes such as parameter tuning.

**References:**

1. Finkel, R.A. and Bentley, J. L. ‘Quad trees a data structure for retrieval on composite keys.’ Actainformatica, 1974; 4: 1–9.

2. 10x Genomics. ‘Preview data: FFPE human lung cancer with xenium multimodal cell segmentation’.

3. Kim N, Kim H.k., Lee K., et al. ‘Single-cell RNA sequencing demonstrates the molecular and cellular reprogramming of metastatic lung adenocarcinoma.’ Nature communications, 2020; 11(1):2285.

4. 10x Genomics. ‘Xenium In Situ: Cell Segmentation’, Technical Note CG000750, Rev A, 2024.